# The Application of Brain Organoids in Assessing Neural Toxicity

**DOI:** 10.3389/fnmol.2022.799397

**Published:** 2022-02-09

**Authors:** Pan Fan, YuanHao Wang, Min Xu, Xiao Han, Yan Liu

**Affiliations:** ^1^State Key Laboratory of Reproductive Medicine, School of Pharmacy, Institute for Stem Cell and Neural Regeneration, Nanjing Medical University, Nanjing, China; ^2^Key Laboratory of Targeted Intervention of Cardiovascular Disease, Collaborative Innovation Center for Cardiovascular Disease Translational Medicine, School of Pharmacy, Nanjing Medical University, Nanjing, China

**Keywords:** brain organoid, neural toxicity, brain organoid transplantation, vascularization, assembloid

## Abstract

The human brain is a complicated and precisely organized organ. Exogenous chemicals, such as pollutants, drugs, and industrial chemicals, may affect the biological processes of the brain or its function and eventually lead to neurological diseases. Animal models may not fully recapitulate the human brain for testing neural toxicity. Brain organoids with self-assembled three-dimensional (3D) structures provide opportunities to generate relevant tests or predictions of human neurotoxicity. In this study, we reviewed recent advances in brain organoid techniques and their application in assessing neural toxicants. We hope this review provides new insights for further progress in brain organoid application in the screening studies of neural toxicants.

## Introduction

The central nervous system (CNS) is the most complex and highly organized organ, is the central control of the body, and coordinates the activities. The CNS is sensitive to exogenous interference, including drugs, environmental pollutants, and food additives. Growing evidence suggests that these perturbations may initiate severe neural toxicity and lead to neurodevelopmental disorders and neurodegenerative disease (Grandjean and Landrigan, 2014; Babadjouni et al., 2017). These disabilities may reduce the quality of life of the patients, disturb their behavior, and then increase the societal and familial burdens (Yamasue and Domes, 2018). Toxicants identified by previous studies are limited, especially those affecting the nervous system. Due to the limitations in the accessibility of human brain tissue and species variation, how these toxicants exert their effects on the nervous system has not been fully elucidated (Crofton et al., 2011; Judson et al., 2014). More efforts need to be put into discovering these toxicants and elaborating how they induce neural toxicity.

Animal models based on rats, mice, rabbits, dogs, and so on are commonly used for *in vivo* neurotoxicity assessment. However, because of species differences between humans and animals, the information generated from animal assays may not be accurate for humans (Chen et al., 2019). In addition, large-scale drug assessment based on animal models lacks efficacy and is time-consuming. The emergence of human pluripotent stem cells (hPSCs), including human embryonic stem cells (hESCs) and human-induced pluripotent stem cells (hiPSCs), provides a new strategy for modeling the developing human brain, neurological disorders (Takahashi et al., 2007; Yu et al., 2007), and neural toxicity assessments (Rowe and Daley, 2019). Conventional two-dimensional (2D) models with uniform cell types lack cell diversity, and they could not fully recapitulate the complex structure of the brain. Neural disorders caused by toxicants occur within the context of a multicellular system, and 2D model assessment systems based on hPSCs could uncover only cell-level phenotypes. Brain organoids, which are three-dimensional (3D) multicellular aggregates generated from hPSCs, could partially recapitulate the structural features of the brain (Dutta et al., 2017). Hence, brain organoid is a potential model for neurotoxicant screening.

Combined with bulk RNA sequences or single-cell RNA sequences, researchers can further analyze the changes between disease and normal organoids at the transcriptional level (Brazovskaja et al., 2019). In this review, we summarized the advances in brain organoid techniques and discussed the application of brain organoids in assessing neural toxicity. We also discussed the limitations of current organoid models and potential improvements.

## Advances in Brain Organoid Techniques

*In vitro* cultured brain organoids have self-organized 3D structures that recapitulate the aspects of neural function (Paşca, 2018). Protocols for generating brain organoids can be classified into two groups: unguided and guided methods (Kelley and Paşca, 2021). Unguided methods are based on intrinsic developmental programs, so brain organoids contain various types of cell lineages (Lancaster et al., 2013; Lancaster and Knoblich, 2014). The unguided brain organoids contain heterogeneous brain region identities, which range from the forebrain, midbrain, and choroid plexus (ChP) to the hindbrain (Quadrato et al., 2017; Makrygianni and Chrousos, 2021). However, because neural induction is stochastic and inconsistent, the brain regions in unguided organoids are positioned randomly, and the same brain regions are not always present in every organoids (Camp et al., 2015; Kelley and Paşca, 2021). Therefore, unguided methods are difficult to fully recapitulate the spatial structure of the brain in a consistent manner, which is the main cause of heterogeneity between batches. In addition, the iPSC lines derived from different individuals also bring heterogeneity because different iPSC lines have different preferences to become certain tissue types under unguided protocols. The heterogeneity also rises from the low surface-area-to-volume ratio. Combining bioengineered constructs into the organoids to arrange cell configuration could increase the reproducibility of unguided brain organoids (Lancaster et al., 2017). Guided methods modulate the morphogen signaling pathways that affect anterior-posterior (A-P) and dorsal-ventral (D-V) patterning to guide brain organoids to obtain specific brain region identity (Tao and Zhang, 2016). Numerous efforts have been made to establish region-specific differentiation protocols. Investigators have already established protocols for constructing region-specific brain organoids, such as the forebrain, midbrain, thalamus, cerebellum, and ChP (Muguruma et al., 2015; Kelava and Lancaster, 2016; Baldassari et al., 2020; Pellegrini et al., 2020b; Xiang et al., 2020). In general, unguided organoids rely on intrinsic pattern signals and could spontaneously generate different brain regions, which are suitable for studies related to modeling the whole brain. Guided organoids with distinct regional identities could be used to answer the questions related to human brain development and disease in the specific brain region.

The extensive application of brain organoids in modeling human nervous system disorders *in vitro* has inspired researchers to improve current brain organoid models and methods. In brief, the major limitations of current brain organoid techniques are nutritional intake in long-term culture and bona fide *in vitro* modeling of the real brain.

### Long-Time Maturation

When organoids become larger after long-term culture, the core of the organoids usually becomes necrotic. The shortage of nutrients and oxygen in the core impedes long-term culture or the formation of more complex structures. Moreover, brain organoids also exhibit elevated cell stress, which is a response to harmful environments, such as hypoxia. The abnormally higher level of cell stress in brain organoids significantly disturbs the developmental procedures in the normal brain (Bhaduri et al., 2020). To solve the abovementioned issues, numerous methods have been established and could partially reduce necrosis in organoids.

Maintaining brain organoids in a spinning bioreactor could enhance nutrient absorption and significantly prolong the survival time (Lancaster et al., 2013). In addition, developing a miniaturized multiwell spinning bioreactor SpinΩ built using the 3D-printing technique increased the throughput and saved cost and space (Qian et al., 2016, 2018). These physical methods provide a dynamic culture system and thus promote the diffusion of nutrients and oxygen to some extent.

Studies have shown that the survival of brain organoids could be prolonged up to 2 years (Gordon et al., 2021). However, due to the lack of *in vivo* circulatory system, the progenitor proliferation is reduced over long-term culture. The insufficiency of nutrients and oxygen in the inner part of brain organoids impedes the maturation and differentiation of neurons and glial cells, which is a major obstacle for maintaining continuous development to maximize brain organoid maturity. Typically, the shortage of nutrients and oxygen in the core of brain organoids is essentially due to *in vitro* cultured brain organoids that lack vasculature (Yin et al., 2016). For this reason, incorporating functional blood vessels into the brain organoids could be a feasible strategy. As endothelial cells are derived from the mesoderm lineage, one strategy is assembling endothelial cells into brain organoids. Inducing endothelial cells (ECs) in brain organoids by treatment or overexpression of human ETS variant 2 (ETV2) contributes to the formation of a complex vascular network in brain organoids (Cakir et al., 2019; Ham et al., 2020). Despite these physical mixing methods, the use of integrated vascular or vascular-like structures could solve this issue to some extent. Furthermore, Mansour et al. (2018) transplanted brain organoids into the mouse brain to allow host circulation to supply the organoid grafts. This is the first study to truly integrate functional vasculature and the bloodstream into the brain organoid system. Similarly, Bhaduri et al. (2020) transplanted brain organoid cells into the mouse brain and quickly reduced the stress to normal levels, and normal developmental procedures resumed. Therefore, brain organoids need *in vivo* environment for maturation.

Another scheme for the same purpose involves integrating human umbilical vein endothelial cells into brain organoids (Shi et al., 2020), adapting the air-liquid interface culture or organ-on-a-chip system (Berger et al., 2018; Wang et al., 2018; Giandomenico et al., 2019), or using the slicing method (Qian et al., 2020).

**FIGURE 1:**
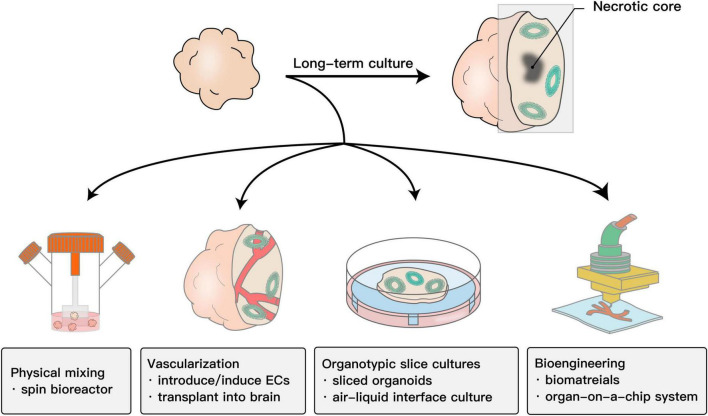
Schematic showing the methods to improve the survival and maturation of brain organoids. Due to the lack of *in vivo* circulatory system, the progenitor proliferation is reduced over long-term culture. The insufficiency of nutrients and oxygen in the inner part of brain organoids impedes the maturation and differentiation of neurons and glial cells. To promote nutrient and oxygen diffusion, physical mixing is the most common method. Adapting bioengineering methods, including vasculature or scaffolds created by 3D printing and organ-on-a-chip systems such as microfluidics, to brain organoid systems also works. In addition, adapting the organotypic cultures into a brain organoid culture system by slicing organoids to reduce the thickness or further culturing them on an air-liquid interface system could also promote the absorption of oxygen and nutrients. The most essential cause of this problem is that brain organoids lack a functional vascular system. Building functional blood vessels could solve this problem to a large extent. Such strategies include introducing/inducting ECs or implanting them into the mouse brain.

All these methods (Figure 1) indeed strongly improved brain organoid survival and promoted functional maturation, thus facilitating the capability of brain organoids to model the human brain. In terms of toxicity evaluation, it allows assessing drug toxicity to CNS in both developmental stage and adults.

### Blood-Cerebrospinal Fluid Barrier Modeling

Cerebrospinal fluid (CSF) can transport nutrients and eliminate by-products in the brain. The ChP can form the blood-CSF barrier (B-CSF-B) and secrete CSF (Lun et al., 2015). Similar to the blood-brain barrier (BBB), the B-CSF-B acts as a barrier to protect the CNS (Ghersi-Egea et al., 2018). In addition to its barrier function, the ChP also plays a role in transporting and metabolizing various nutrients, hormones, and other compounds (Ghersi-Egea et al., 2018). In pharmacology and toxicology studies, *in vitro* CNS barrier models are critical for testing whether therapeutic drugs could reach the CNS and exert a therapeutic or toxic effect. However, modeling a functional brain barrier system remains a challenge for current organoid techniques. Organoids generated by assembling endothelial cells, pericytes, and astrocytes express tight junctions, molecular transporters, and drug efflux pumps, which present the main features of the BBB (Cho et al., 2017; Bergmann et al., 2018). In addition to the BBB, Pellegrini et al. (2020b) generated ChP organoids that formed tight junctions and could secrete CSF-like fluid. ChP organoids have been proven to qualitatively and quantitatively predict the permeability of drugs and have been successfully applied to demonstrate that ChP cells are the main target of SARS-CoV-2 virus in the CNS (Jacob et al., 2020; Pellegrini et al., 2020a). Hence, the generation of brain organoids containing brain barrier-like structures offers a reliable model for disease modeling and investigates the neurotoxicity of drugs and potential mechanisms.

### Assembloids

The human brain is composed of various regions arranged in spatial order. Normal brain functions rely on interregional interactions based on neural circuits between brain regions. Although brain organoids have been employed to model certain aspects of the brain, neither guided nor unguided methods could model interactions between brain regions. Generating organoids containing ordered multi-region brain organoids is, therefore, necessary. To model the interregional interactions, several groups developed methods, including introducing a signal center or fusing organoids of different regions. Sonic hedgehog (SHH) guides D-V patterning in a concentration gradient-dependent manner (Tao and Zhang, 2016). Introducing an SHH signal center into one pole of the developing forebrain organoid provided an asymmetric morphogenetic cue for generating *in vivo* topography-like brain organoids (Cederquist et al., 2019). The concept of modularity was also introduced into modeling multiple regions of the brain with brain organoids. Specifically, generating different brain region-specific organoids separately and then fusing them together could model complex interactions between brain regions (Bagley et al., 2017; Birey et al., 2017). Fused medial ganglionic eminence (MGE) and cortical organoids could recapitulate the interneuron migration between MGE and the cortex, which provided deeper insights into molecular dynamics during human brain development (Xiang et al., 2017). The fused corticothalamic organoids and cortico-striatal organoids could model axon projections between the thalamus or striatum and cortex, which allowed us to investigate circuit connectivity development and related disorders (Xiang et al., 2019; Miura et al., 2020). In addition, building cortical-motor assembloids by fusing cortical, spinal, and skeletal muscle spheroids generates corticomotor circuits. In these assembloids, activating cortical neurons could control muscle contraction by activating motor neurons, which provides tools for investigating the cortico-spinal-muscle circuit (Andersen et al., 2020). Fused brain organoids containing prepatterned region identities could recapitulate interregional interactions and, thereby, have great potential in studying human brain nervous system disorders (Chen et al., 2020).

### Brain Organoid Transplantation

The capacity to repair and replace injured neurons in the adult brain is limited (Sun, 2016). Previous treatment of neurodegeneration and CNS injury is mainly slowing down the neuronal damage and decreasing the loss of neural cells. The neural transplantation strategy aimed at supplementing damaged/lost neurons to promote functional repair of brain lesions brings promise for the treatment of these diseases. Transplanted region-specific progenitor cells could differentiate into certain types of cells and integrate into damaged areas. Indeed, transplantation of hPSC-derived defined neurons into the brain focal zone of rodent and non-human primate models has shown improvements in damaged functions (Kriks et al., 2011; Kikuchi et al., 2017; Tao et al., 2021). Since brain organoids contain various neuronal cell types, brain organoid transplantation may provide another option to repair large lesions in the brain. However, whether brain organoids could be utilized in transplantation is not clear.

Mansour et al. (2018) transplanted brain organoids into the mouse brain to facilitate vascularization. Compared with NSC transplantation, organoid transplantation showed increased cell survival, progressive neuronal differentiation, and maturation and formation of robust vascularization and neuronal networks with the host brain (Daviaud et al., 2018; Mansour et al., 2018). Our group generated small human cerebral organoids and transplanted them into the mouse medial prefrontal cortex (mPFC) (Dong et al., 2021). The grafts established subcortical projections and formed mutual synaptic connections with host mouse neurons. In addition, the host mice demonstrated an increased startle fear response, suggesting the functional integration of grafts in the mouse brain (Dong et al., 2021). Brain organoids consist of various neural cell types and are sufficient for repairing brain injury. Therefore, in a microenvironment that promotes better survival after transplantation, brain organoids, due to their advantages in cell-type diversity and modeling mini-brain tissue, may become an optional source of neural repair in large-scale injury, such as stroke and brain injury (Wang et al., 2020).

Furthermore, in addition to prospects in repair treatment, transplanting brain organoids into the mouse brain may also be a potential tool for investigating the impacts of neural toxicants on neural migration and projection. Specifically, human organoids, including neurons and glial cells, integrate into host brains after implantation. The *in vivo* environment promotes the maturation of both the morphology and function of neurons and glial cells. This human-mouse chimeric brain model could be further applied to test how the compounds affect human neurons dynamically after physiological absorption and distribution processes, including the process of crossing the BBB.

Together, brain organoid technologies provide a robust platform for the *in vitro* studies of human brain development, neuronal function, and disorders. Although the technology has recently made great progress, many deficiencies remain to be further optimized.

## Application of Brain Organoids in Assessing Neural Toxicity

The CNS is sensitive to exogenous interference, and perturbations in exogenous chemicals could disturb the normal developmental process or function of the brain, ultimately resulting in nervous system disorders. Experiments based on animal models have provided abundant information about neural toxicants, but due to species differences, part of the information generated from animal assays may not always be accurate for humans (Hartung, 2009). Moreover, animal models are usually not suitable for large-scale screening because of the labor and time costs (Hou et al., 2013).

The hPSCs are capable of generating different cell types and, therefore, offer a platform for identifying toxicants *in vitro*. Studies have verified the feasibility of hiPSCs for predicting neural toxicity by validating the effects of well-known toxicants, such as retinoic acid (Colleoni et al., 2011) and ethanol (Kim et al., 2014, 2016), on the brain. These studies identified genes that participate in neural toxicity processes and thus paved the way for identifying neural toxicants on a large scale. Importantly, neural toxicants may affect multiple cell types in the brain, while the 2D monolayer culture system could not mimic the complex structure and the inherent physiological conditions of the brain (Gupta et al., 2016). The 3D brain organoids contain diverse types of cells and exhibit similar brain structures; thus, brain organoids may have great potential to bridge unfilled gaps.

Compared with animal models, *in vitro* studies have revealed that cultured brain organoids have more similarities in cell composition, gene expression profiles, and protein composition with human fetal neocortex (Camp et al., 2015; Bershteyn et al., 2017; Nascimento et al., 2019). For example, brain organoids have the characteristics of the outer subventricular zone (oSVZ), while mouse brains do not have this layer (Smart et al., 2002; Lui et al., 2011; Lancaster et al., 2013). oSVZ progenitors contain radial glial cells, which are one of the main contributors to neurogenesis and cortical expansion in the human brain (Fietz et al., 2010). Therefore, alteration of oSVZ could be used as the potential readout for the effects of neurotoxicants on the developing brain. Taken together, brain organoids have provided high-performance and high-throughput platforms to assess neural toxicants.

### Application in Assessing Neural Toxicants

With the rapid development of society, humans are actively or passively exposed to various chemicals. During gestation, prenatal exposure to these chemicals may result in severe embryonic malformation or nervous system disabilities, including but not limited to microcephaly, facial malformation, autism, attention-deficit hyperactivity disorder, and other nervous system disorders (Grandjean and Landrigan, 2014). Because of species variation and ethical issues, it is not easy to explore how these chemicals induce abnormal embryonic development in animal models or preclinical trials. For example, thalidomide was used to treat sickness for pregnant women and, unfortunately, it caused limb hypoplasia in fetuses (Franks et al., 2004; Knobloch and Rüther, 2008). These incidents were attributed to differences in the CYP3A7 enzyme between humans and rodents (Kazuki et al., 2016). Thus, the use of animal models in drug development stages failed to anticipate these incidents. Conclusions obtained from animal assays may not be applicable to humans, so the results need further validation in humans, especially for the toxicity.

Human brain organoids provide a promising strategy for neural toxicity research *in vitro*. The most well-known *in vitro* embryotoxicity test system is the embryonic stem cell test (EST) (Seiler and Spielmann, 2011). ESCs have been utilized to assess embryotoxicants at different time periods. Similar to the EST, the process of evaluating neurotoxic substances in brain organoids typically involves adding compounds to the brain organoid culture system at a certain time to simulate acute or prolonged exposure during pregnancy and then analyzing the morphological, transcriptional, and functional changes.

Drug abuse or substance abuse is increasing at alarming rates, which has caused serious social problems. Previous studies have revealed that prenatal exposure to cocaine could disrupt cortical development and ultimately impair neural behavioral development (Singer et al., 2002; Rando et al., 2013). Studies in rodents demonstrated that prenatal cocaine exposure induces changes related to CYP-mediated drug metabolism in the cytoarchitecture of the embryonic neocortex (Lee et al., 2011). Nevertheless, the significant differences in neocorticogenesis and CYP enzymes between humans and rodents make it difficult to translate findings from rodents to humans. Because CYP3A5 is predominantly expressed at the early stage of neocorticogenesis, Lee et al. (2017) employed brain organoids to model prenatal cocaine exposure. Cocaine was added to the culture at a concentration of 3 μM for 1 h every other day. The researchers demonstrated that cocaine induces brain development deficits through CYP3A5-mediated reactive oxygen species generation, neocortical progenitor cell proliferation inhibition, induction of premature neuronal differentiation, and interruption of neural tissue development. Treatments targeting CYP3A5 could alleviate the nervous system deficits caused by prenatal cocaine exposure in humans. Methamphetamine (METH) is a stimulant that causes system-wide changes to the brain when abused. Clinical studies have shown that prenatal METH exposure could cause fetal growth restriction (Smith et al., 2006; Nguyen et al., 2010). Dang et al. (2020) utilized brain organoids to study the effect of prenatal METH exposure on the developing brain. They treated cerebral organoids with 5 μM METH, which is a physiologically relevant concentration. They then confirmed that METH treatment induced gliosis and neuroinflammation, ultimately resulting in neurotoxicity. Using single-cell sequencing technology, they observed robust transcriptional responses in METH-treated glial cell types, which were often overlooked in other studies. Similar to drugs, prenatal alcohol exposure could also induce brain abnormities at both the biochemical and structural levels, which is known as fetal alcohol spectrum disorder (FASD). Insufficiently, current studies mostly focused on the second and third trimesters, and few studies have addressed the first trimester (Riley and McGee, 2005; O’Connor and Paley, 2009). Several studies employed brain organoids to investigate the effects of alcohol exposure on early developmental stages (Zhu et al., 2017; Arzua et al., 2020). The ethanol concentrations in these studies were equivalent to or below the blood alcohol concentration of binge drinking studies. These researchers found that prenatal alcohol exposure could lead to reduced cell proliferation, impaired neural differentiation, increased cell death, and extensive transcriptomic changes. Therefore, brain organoids offer a feasible model for neurotoxicity studies.

Active exposure is based on personal initiative, while passive exposure usually happens without realizing it, such as second-hand smoking and air pollution. In contrast to active exposure, passive exposure to ubiquitous environmental chemicals cannot be avoided, which is individually reported to be associated with adverse pregnancy outcomes (Bellinger, 2013; Padula et al., 2020). Investigators harnessed brain organoids to estimate the link between environmental chemical exposure and nervous system disorders. For example, nicotine exposure induced premature neuronal differentiation and damaged cortical development (Qiao et al., 2018), and valproic acid exposure caused nervous system dysfunction and increased the risk of autism (Cui et al., 2020). The difference between active and passive exposure is that the concentration that is absorbed into the body would be not the same. In addition, the duration also varies substantially. Ultimately, the degree of impact on the fetus is not the same.

Numerous studies have shown that exposure to environmental toxicants during pregnancy might contribute to the occurrence of nervous system disorders (Pamies et al., 2018). However, the majority of environmental toxicants are undefined. Brain organoids could be used to establish an efficient and reliable platform for screening environmental chemicals that are toxic to neurodevelopment. Investigators have indeed tried to establish such a platform and tested the feasibility of some known toxicants (Sandström et al., 2017).

These findings support that brain organoids offer a reliable tool for illustrating the mechanism underlying nervous system disorders caused by toxicants. The combination of brain organoid techniques, machine learning, and omics data enables a platform for screening neural toxicants on a large scale.

### Application in High-Throughput Toxicity Testing

Chemical compounds are widely used in human life, while safety testing results from *in vitro* or animal tests are usually difficult to verify in humans because of practical and ethical constraints (Parasuraman, 2011). Brain organoids, which resemble human tissues and are cultured in dishes, provide the opportunity to generate more relevant predictions for human neurotoxicity (Truskey, 2018; Takahashi, 2019). Building a toxicity testing system to identify such compounds before their widespread application may reduce the potential for disease.

It would be challenging to combine brain organoids with high-throughput methods for toxicity screening because efficient testing of a large number of compounds at various concentrations requires automated high-throughput workflows, which challenges the capacity and practicality of a model. Renner et al. (2021) developed a highly homogeneous and reproducible 3D model system of the human midbrain for high-throughput screening applications. They verified the capability of high-throughput screening by applying this system to assess the general neurotoxic and dopaminergic neuron-specific toxic effects of a library of 84 compounds, and they demonstrated the feasibility of quantitatively assessing cell-type-specific toxicity in human organoids. In addition to neural functional analysis, Sirenko et al. (2019) employed calcium oscillations as a readout to evaluate neurotoxicity, as calcium signaling is one aspect of capturing the effects related to neurotoxicity.

**FIGURE 2:**
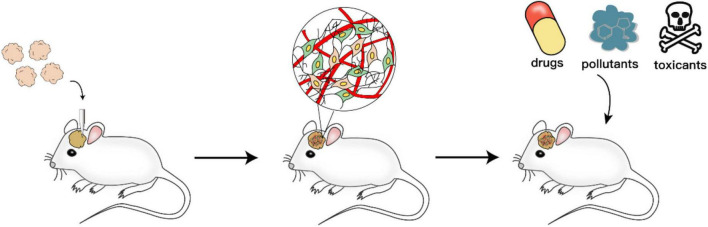
Human-mouse chimeric brain in neurotoxicity study. Brain organoids are transplanted into the mouse brain to establish a human-mouse chimeric brain model. Two to three months after human cells are incorporated into the host brain, the test compounds are administered to the mice. Then, subsequent readouts of neurotoxicity could be obtained by behavioural tests, immunohistochemistry, electrophysiology and other experiments.

Brain organoids could be applied as a robust high-throughput platform for screening environmental toxicants (Figure 2). Combining morphological analysis, functional evaluation, multiomics data, and machine learning algorithms enables a comprehensive and reliable high-throughput screening of environmental toxicants. Furthermore, these high-throughput models also have utility for drug safety evaluation.

## Further Directions

In recent years, there have been breakthroughs not only in brain organoid generation technology but also in the application in many aspects, such as disease modeling, toxicity screening, and drug screening. Brain organoids have validated their value as a model for the human brain, even though it is still difficult to fully recapitulate the real human brain. First, the cell-type diversity needs further expansion in brain organoids (Sun et al., 2021). Although protocols have been established to enrich neuronal cell types in brain organoids, including non-neuronal lineage cells such as endothelial cells and microglial cells (Abud et al., 2017; Cakir et al., 2019; Ham et al., 2020; Shi et al., 2020), it is still difficult to establish the immune environment in brain organoids. Thus, current brain organoids could not be used to fully model the interactions among brain tissues, i.e., the vasculature and inflammatory components (Pham et al., 2018; Chukwurah et al., 2019; Fagerlund et al., 2021). Future studies need to focus on importing non-neural cell components and integrating bioengineering approaches to construct brain organoids that include flowing blood or blood substitutes and immunologic niches.

Although brain organoids could mimic the human brain to some degree, brain organoids cannot generate the same pattern as the brain due to the lack of supportive tissue and body axes (Wang, 2018). The current brain organoid protocols usually depend on the inherent nature of self-organization into distinct structures, which results in heterogeneity and batch effects. Large-scale toxicant screening studies would be disturbed in the acquisition of conclusive and generalizable results (Costamagna et al., 2021). Reducing heterogeneity is essential for the application of brain organoids in disease modeling and large-scale studies. Optimizing the early steps of brain organoid generation protocols, such as embryoid formation and neural induction, could facilitate the production of uniform human brain organoids (Sivitilli et al., 2020). Brain organoids typically contain dozens of neural tubes, and the number of neural tubes in each organoid is different, which leads to heterogeneity in the size of brain organoids. Therefore, generating brain organoids containing a single neural tube might ensure the production of uniform brain organoids (Wang et al., 2021). However, current methods are based on manual isolation of a single neural tube, which is still labor consuming. In addition, adopting more engineering strategies into brain organoid generating systems could reduce heterogeneity to a large extent (Hofer and Lutolf, 2021). These strategies include increasing the degree of automation, using defined media, and performing evaluations in real time.

To model the human brain development process and elaborate structure, brain organoids have the potential to predict compound effects on the CNS. Studies have employed brain organoids to investigate the mechanism of neurodevelopmental disorders caused by prenatal exposure to specific chemicals. Combining morphological analysis, functional evaluation, multiomics data, and machine learning algorithms facilitates organoid-based screening platforms that work in a high-throughput and high-performance manner. However, it must be pointed out that strategies based on self-organization and default-differentiation protocols may lead to data variation, so the differentiation process should be strictly controlled. Generating region-specific organoids, such as utilizing midbrain organoids to assess cell-type-specific toxicity, would to some extent reduce the variants. In addition, readouts are usually obtained by optical sensors, which could provide only limited information (Hofer and Lutolf, 2021). Integrating multiple types of sensors, such as MEAs and microcameras, into the brain organoid system to perform real-time detection is challenging and requires miniaturized sensors and relevant adjustment of the culture system.

**FIGURE 3:**
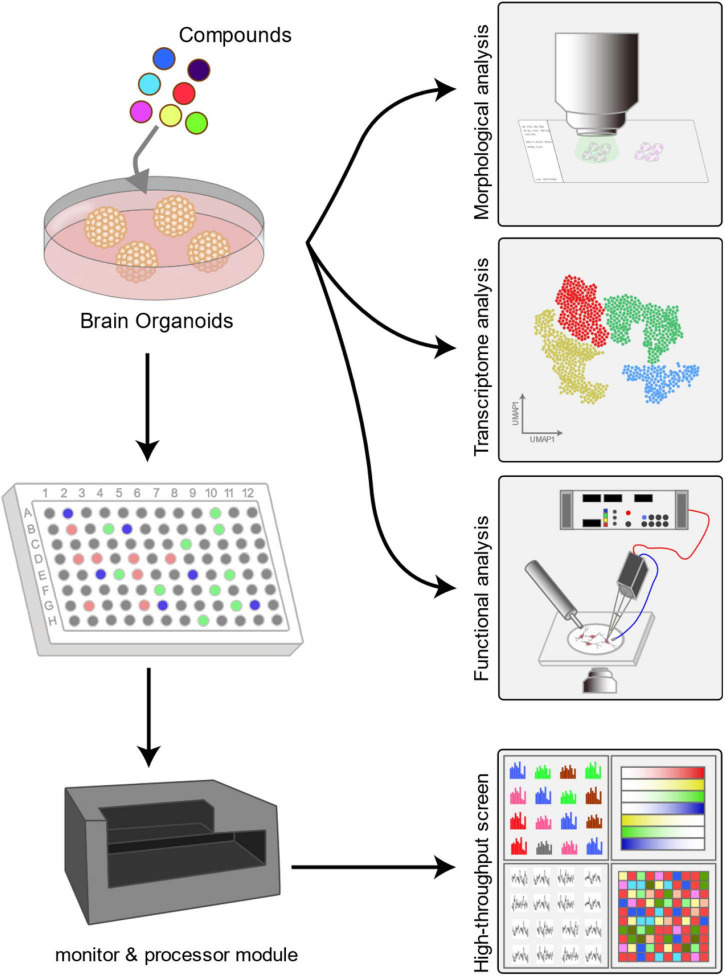
Application of brain organoids in neurotoxicity assessment. Typically, neurotoxicity to brain organoids is measured by morphological analysis, such as bright field microscopy and immunohistochemical analysis. By combining transcriptomics, metabolomics and proteomics analysis, researchers could identify the certain mechanisms underlying neurotoxicity. Additionally, the functional changes in neurons can be clarified by electrophysiology and calcium imaging analysis. Moreover, in high-throughput neurotoxicity studies, the readouts are usually collected by miniaturized monitors such as MEAs and microcameras and then processed by a machine learning algorithm processor. The combination of machine learning algorithms enables comprehensive and reliable high-throughput neurotoxicity assessment.

Although numerous studies have proven that brain organoids have great potential in neurotoxicant screening and further mechanistic studies, animal studies are still essential. Brain organoids which are human-derived cells have the advantages of *in vitro* modeling of the human brain development process, and a relatively short experimental period could be applied to high-throughput screening studies. However, the disadvantages are also obvious. The current brain organoid technologies are not capable of modeling functional placentas in the brain organoid system. Thus, brain organoids could be used only as a preliminary assessment tool before animal studies or as an added validation tool after animal experiments. In addition, almost all the tested compounds in brain organoids are applied directly to the medium, which is convenient but ignores the process of absorption and distribution. Hence, the obtained conclusions need to be validated in animal experiments. Alternatively, the human-mouse chimeric brain could be used to test how compounds affect neuronal function after physiological absorption and distribution processes, including the process of crossing the BBB (Figure 3). Nevertheless, the current brain organoid technique acts as a prescreening or verification tool in neurotoxicity research. All these shortcomings of brain organoid techniques require further improvement for more realistic recapitulation of the real *in vivo* environment.

In conclusion, the brain organoid technique provides a robust tool for evaluating the toxic compound exposure in nervous system diseases. A high-throughput neural toxicant screening platform allows the screening of possible neural toxicants before their widespread use and thus reduces the risk of potential disease. Although inadequacies still exist in the current brain organoid techniques, recent studies have widely demonstrated that brain organoids hold great promise in neural toxicant examinations. With the development of bioengineering and automation techniques, brain organoids would be generated in batches with uniform properties to facilitate neural toxicant assessment studies.

## Author Contributions

YL and XH conceived of the presented idea. PF and MX prepared the draft manuscript. YW revised the manuscript. All authors listed have made a substantial, direct, and intellectual contribution to the work, and approved it for publication.

## Conflict of Interest

The authors declare that the research was conducted in the absence of any commercial or financial relationships that could be construed as a potential conflict of interest.

## Publisher’s Note

All claims expressed in this article are solely those of the authors and do not necessarily represent those of their affiliated organizations, or those of the publisher, the editors and the reviewers. Any product that may be evaluated in this article, or claim that may be made by its manufacturer, is not guaranteed or endorsed by the publisher.

## References

[B1] Abud E. M. Ramirez R. N. Martinez E. S. Healy L. M. Nguyen C. H. H. Newman S. A. (2017). iPSC-Derived Human Microglia-like Cells to Study Neurological Diseases. *Neuron* 94 278.e–293.e. 10.1016/j.neuron.2017.03.042 28426964 PMC5482419

[B2] Andersen J. Revah O. Miura Y. Thom N. Amin N. D. Kelley K. W. (2020). Generation of Functional Human 3D Cortico-Motor Assembloids. *Cell* 183 1913.e–1929.e. 10.1016/j.cell.2020.11.017 33333020 PMC8711252

[B3] Arzua T. Yan Y. Jiang C. Logan S. Allison R. L. Wells C. (2020). Modeling alcohol-induced neurotoxicity using human induced pluripotent stem cell-derived three-dimensional cerebral organoids. *Transl. Psychiatry.* 10:347. 10.1038/s41398-020-01029-4 33051447 PMC7553959

[B4] Babadjouni R. M. Hodis D. M. Radwanski R. Durazo R. Patel A. Liu Q. (2017). Clinical effects of air pollution on the central nervous system; a review. *J. Clin. Neurosci.* 43 16–24. 10.1016/j.jocn.2017.04.028 28528896 PMC5544553

[B5] Bagley J. A. Reumann D. Bian S. évi-Strauss J. L. Knoblich J. A. (2017). Fused cerebral organoids model interactions between brain regions. *Nat. Methods* 14 743–751. 10.1038/nmeth.4304 28504681 PMC5540177

[B6] Baldassari S. Musante I. Iacomino M. Zara F. Salpietro V. Scudieri P. (2020). Brain Organoids as Model Systems for Genetic Neurodevelopmental Disorders. *Front. Cell. Dev. Biol.* 8:590119. 10.3389/fcell.2020.590119 33154971 PMC7586734

[B7] Bellinger D. C. (2013). Prenatal Exposures to Environmental Chemicals and Children’s Neurodevelopment: An Update. *Saf. Health Work* 4 1–11. 10.5491/SHAW.2013.4.1.1 23515885 PMC3601292

[B8] Berger E. Magliaro C. Paczia N. Monzel A. S. Antony P. Linster C. L. (2018). Millifluidic culture improves human midbrain organoid vitality and differentiation. *Lab. Chip.* 18 3172–3183. 10.1039/c8lc00206a 30204191

[B9] Bergmann S. Lawler S. E. Qu Y. Fadzen C. M. Wolfe J. M. Regan M. S. (2018). Blood-brain-barrier organoids for investigating the permeability of CNS therapeutics. *Nat. Protoc.* 13 2827–2843. 10.1038/s41596-018-0066-x 30382243 PMC6673652

[B10] Bershteyn M. Nowakowski T. J. Pollen A. A. Di Lullo E. Nene A. Wynshaw-Boris A. (2017). Human iPSC-Derived Cerebral Organoids Model Cellular Features of Lissencephaly and Reveal Prolonged Mitosis of Outer Radial Glia. *Cell Stem Cell* 20 435.e–449.e. 10.1016/j.stem.2016.12.007 28111201 PMC5667944

[B11] Bhaduri A. Andrews M. G. Mancia Leon W. Jung D. Shin D. Allen D. (2020). Cell stress in cortical organoids impairs molecular subtype specification. *Nature* 578 142–148. 10.1038/s41586-020-1962-0 31996853 PMC7433012

[B12] Birey F. Andersen J. Makinson C. D. Islam S. Wei W. Huber N. (2017). Assembly of functionally integrated human forebrain spheroids. *Nature* 545 54–59. 10.1038/nature22330 28445465 PMC5805137

[B13] Brazovskaja A. Treutlein B. Camp J. G. (2019). High-throughput single-cell transcriptomics on organoids. *Curr. Opin. Biotechnol.* 55 167–171. 10.1016/j.copbio.2018.11.002 30504008

[B14] Cakir B. Xiang Y. Tanaka Y. Kural M. H. Parent M. Kang Y. J. (2019). Engineering of human brain organoids with a functional vascular-like system. *Nat. Methods* 16 1169–1175. 10.1038/s41592-019-0586-5 31591580 PMC6918722

[B15] Camp J. G. Badsha F. Florio M. Kanton S. Gerber T. Wilsch-Bräuninger M. (2015). Human cerebral organoids recapitulate gene expression programs of fetal neocortex development. *Proc. Natl. Acad. Sci. U.S.A* 112 15672–15677. 10.1073/pnas.1520760112 26644564 PMC4697386

[B16] Cederquist G. Y. Asciolla J. J. Tchieu J. Walsh R. M. Cornacchia D. Resh M. D. (2019). Specification of positional identity in forebrain organoids. *Nat. Biotechnol.* 37 436–444. 10.1038/s41587-019-0085-3 30936566 PMC6447454

[B17] Chen A. Guo Z. Fang L. Bian S. (2020). Application of Fused Organoid Models to Study Human Brain Development and Neural Disorders. *Front. Cell. Neurosci.* 14:133. 10.3389/fncel.2020.00133 32670022 PMC7326106

[B18] Chen H. I. Song H. Ming G. L. (2019). Applications of Human Brain Organoids to Clinical Problems. *Dev. Dyn.* 248 53–64. 10.1002/dvdy.24662 30091290 PMC6312736

[B19] Cho C. F. Wolfe J. M. Fadzen C. M. Calligaris D. Hornburg K. Chiocca E. A. (2017). Blood-brain-barrier spheroids as an in vitro screening platform for brain-penetrating agents. *Nat. Commun.* 8:15623. 10.1038/ncomms15623 28585535 PMC5467173

[B20] Chukwurah E. Osmundsen A. Davis S. W. Lizarraga S. B. (2019). All Together Now: Modeling the Interaction of Neural With Non-neural Systems Using Organoid Models. *Front. Neurosci.* 13:582. 10.3389/fnins.2019.00582 31293366 PMC6598414

[B21] Colleoni S. Galli C. Gaspar J. A. Meganathan K. Jagtap S. Hescheler J. (2011). Development of a neural teratogenicity test based on human embryonic stem cells: response to retinoic acid exposure. *Toxicol. Sci.* 124 370–377. 10.1093/toxsci/kfr245 21934132

[B22] Costamagna G. Comi G. P. Corti S. (2021). Advancing Drug Discovery for Neurological Disorders Using iPSC-Derived Neural Organoids. *Int. J. Mol. Sci.* 22:2659. 10.3390/ijms22052659 33800815 PMC7961877

[B23] Crofton K. M. Mundy W. R. Lein P. J. Bal-Price A. Coecke S. Seiler A. E. (2011). Developmental neurotoxicity testing: recommendations for developing alternative methods for the screening and prioritization of chemicals. *Altex* 28 9–15. 21311847

[B24] Cui K. Wang Y. Zhu Y. Tao T. Yin F. Guo Y. (2020). Neurodevelopmental impairment induced by prenatal valproic acid exposure shown with the human cortical organoid-on-a-chip model. *Microsyst. Nanoeng.* 6:49. 10.1038/s41378-020-0165-z 34567661 PMC8433196

[B25] Dang J. Tiwari S. K. Agrawal K. Hui H. Qin Y. Rana T. M. (2020). Glial cell diversity and methamphetamine-induced neuroinflammation in human cerebral organoids. *Mol. Psychiatry*. 26 1194–1207. 10.1038/s41380-020-0676-x 32051547 PMC7423603

[B26] Daviaud N. Friedel R. H. Zou H. (2018). Vascularization and Engraftment of Transplanted Human Cerebral Organoids in Mouse Cortex. *eNeuro* 5 ENEURO.219–ENEURO.218. 10.1523/eneuro.0219-18.2018 30460331 PMC6243198

[B27] Dong X. Xu S. B. Chen X. Tao M. Tang X. Y. Fang K. H. (2021). Human cerebral organoids establish subcortical projections in the mouse brain after transplantation. *Mol. Psychiatry.* 26 2964–2976. 10.1038/s41380-020-00910-4 33051604 PMC8505255

[B28] Dutta D. Heo I. Clevers H. (2017). Disease Modeling in Stem Cell-Derived 3D Organoid Systems. *Trends. Mol. Med.* 23 393–410. 10.1016/j.molmed.2017.02.007 28341301

[B29] Fagerlund I. Dougalis A. Shakirzyanova A. ómez-Budia M. G. Konttinen H. Ohtonen S. (2021). Microglia orchestrate neuronal activity in brain organoids. biorxiv [preprint] 37737898. 10.3390/cells9112434

[B30] Fietz S. A. Kelava I. Vogt J. Wilsch-Bräuninger M. Stenzel D. Fish J. L. (2010). OSVZ progenitors of human and ferret neocortex are epithelial-like and expand by integrin signaling. *Nat. Neurosci.* 13 690–699. 10.1038/nn.2553 20436478

[B31] Franks M. E. Macpherson G. R. Figg W. D. (2004). Thalidomide. *Lancet* 363 1802–1811. 15172781 10.1016/S0140-6736(04)16308-3

[B32] Ghersi-Egea J.-F. Strazielle N. Catala M. Silva-Vargas V. Doetsch F. Engelhardt B. (2018). Molecular anatomy and functions of the choroidal blood-cerebrospinal fluid barrier in health and disease. *Acta. Neuropathologica.* 135 337–361. 10.1007/s00401-018-1807-1 29368213

[B33] Giandomenico S. L. Mierau S. B. Gibbons G. M. Wenger L. M. D. Masullo L. Sit T. (2019). Cerebral organoids at the air-liquid interface generate diverse nerve tracts with functional output. *Nat. Neurosci.* 22 669–679. 10.1038/s41593-019-0350-2 30886407 PMC6436729

[B34] Gordon A. Yoon S. J. Tran S. S. Makinson C. D. Park J. Y. Andersen J. (2021). Long-term maturation of human cortical organoids matches key early postnatal transitions. *Nat. Neurosci.* 24 331–342. 10.1038/s41593-021-00802-y 33619405 PMC8109149

[B35] Grandjean P. Landrigan P. J. (2014). Neurobehavioural effects of developmental toxicity. *Lancet Neurol.* 13 330–338. 10.1016/s1474-4422(13)70278-3 24556010 PMC4418502

[B36] Gupta N. Liu J. R. Patel B. Solomon D. E. Vaidya B. Gupta V. (2016). Microfluidics-based 3D cell culture models: utility in novel drug discovery and delivery research. *Bioeng. Transl. med.* 1 63–81. 10.1002/btm2.10013 29313007 PMC5689508

[B37] Ham O. Jin Y. B. Kim J. Lee M. O. (2020). Blood vessel formation in cerebral organoids formed from human embryonic stem cells. *Biochem. Biophys. Res. Commun.* 521 84–90. 10.1016/j.bbrc.2019.10.079 31629471

[B38] Hartung T. (2009). Toxicology for the twenty-first century. *Nature* 460 208–212. 10.1038/460208a 19587762

[B39] Hofer M. Lutolf M. P. (2021). Engineering organoids. *Nat. Rev. Mat.* 6 402–420. 10.1038/s41578-021-00279-y 33623712 PMC7893133

[B40] Hou Z. Zhang J. Schwartz M. P. Stewart R. Page C. D. Murphy W. L. (2013). A human pluripotent stem cell platform for assessing developmental neural toxicity screening. *Stem. Cell. Res. Ther.* 1(Suppl. 1):S12. 10.1186/scrt373 24565336 PMC3983661

[B41] Jacob F. Pather S. R. Huang W. K. Zhang F. Wong S. Z. H. Zhou H. (2020). Human Pluripotent Stem Cell-Derived Neural Cells and Brain Organoids Reveal SARS-CoV-2 Neurotropism Predominates in Choroid Plexus Epithelium. *Cell Stem Cell* 27 937.e–950.e. 10.1016/j.stem.2020.09.016 33010822 PMC7505550

[B42] Judson R. Houck K. Martin M. Knudsen T. Thomas R. S. Sipes N. (2014). In vitro and modelling approaches to risk assessment from the U.S. Environmental Protection Agency ToxCast programme. *Basic Clin. Pharmacol. Toxicol.* 115 69–76. 10.1111/bcpt.12239 24684691

[B43] Kazuki Y. Akita M. Kobayashi K. Osaki M. Satoh D. Ohta R. (2016). Thalidomide-induced limb abnormalities in a humanized CYP3A mouse model. *Sci. Rep.* 6:21419. 10.1038/srep21419 26903378 PMC4763305

[B44] Kelava I. Lancaster M. A. (2016). Stem Cell Models of Human Brain Development. *Cell Stem Cell* 18 736–748. 10.1016/j.stem.2016.05.022 27257762

[B45] Kelley K. W. Paşca S. P. (2021). Human brain organogenesis: Toward a cellular understanding of development and disease. *Cell* 185 42–61. 10.1016/j.cell.2021.10.003 34774127 PMC13523617

[B46] Kikuchi T. Morizane A. Doi D. Magotani H. Onoe H. Hayashi T. (2017). Human iPS cell-derived dopaminergic neurons function in a primate Parkinson’s disease model. *Nature* 548 592–596. 10.1038/nature23664 28858313

[B47] Kim J. J. Duan L. Tu T. G. Elie O. Kim Y. Mathiyakom N. (2014). Molecular effect of ethanol during neural differentiation of human embryonic stem cells in vitro. *Genom. Data* 2 139–143. 10.1016/j.gdata.2014.06.012 25089259 PMC4114725

[B48] Kim Y. Y. Roubal I. Lee Y. S. Kim J. S. Hoang M. Mathiyakom N. (2016). Alcohol-Induced Molecular Dysregulation in Human Embryonic Stem Cell-Derived Neural Precursor Cells. *PLoS One* 11:e0163812. 10.1371/journal.pone.0163812 27682028 PMC5040434

[B49] Knobloch J. Rüther U. (2008). Shedding light on an old mystery: thalidomide suppresses survival pathways to induce limb defects. *Cell Cycle* 7 1121–1127. 10.4161/cc.7.9.5793 18418038

[B50] Kriks S. Shim J.-W. Piao J. Ganat Y. M. Wakeman D. R. Xie Z. (2011). Dopamine neurons derived from human ES cells efficiently engraft in animal models of Parkinson’s disease. *Nature* 480 547–551. 10.1038/nature10648 22056989 PMC3245796

[B51] Lancaster M. A. Corsini N. S. Wolfinger S. Gustafson E. H. Phillips A. W. Burkard T. R. (2017). Guided self-organization and cortical plate formation in human brain organoids. *Nat. Biotechnol.* 35 659–666. 10.1038/nbt.3906 28562594 PMC5824977

[B52] Lancaster M. A. Knoblich J. A. (2014). Organogenesis in a dish: modeling development and disease using organoid technologies. *Science* 345:1247125. 10.1126/science.1247125 25035496

[B53] Lancaster M. A. Renner M. Martin C. A. Wenzel D. Bicknell L. S. Hurles M. E. (2013). Cerebral organoids model human brain development and microcephaly. *Nature* 501 373–379. 10.1038/nature12517 23995685 PMC3817409

[B54] Lee C. T. Chen J. Kindberg A. A. Bendriem R. M. Spivak C. E. Williams M. P. (2017). CYP3A5 Mediates Effects of Cocaine on Human Neocorticogenesis: Studies using an In Vitro 3D Self-Organized hPSC Model with a Single Cortex-Like Unit. *Neuropsychopharmacology* 42 774–784. 10.1038/npp.2016.156 27534267 PMC5240177

[B55] Lee C. T. Chen J. Worden L. T. Freed W. J. (2011). Cocaine causes deficits in radial migration and alters the distribution of glutamate and GABA neurons in the developing rat cerebral cortex. *Synapse* 65 21–34. 10.1002/syn.20814 20506319 PMC2965825

[B56] Lui J. H. Hansen D. V. Kriegstein A. R. (2011). Development and evolution of the human neocortex. *Cell* 146 18–36. 10.1016/j.cell.2011.06.030 21729779 PMC3610574

[B57] Lun M. P. Monuki E. S. Lehtinen M. K. (2015). Development and functions of the choroid plexus–cerebrospinal fluid system. *Nat. Rev. Neurosci.* 16 445–457. 10.1038/nrn3921 26174708 PMC4629451

[B58] Makrygianni E. A. Chrousos G. P. (2021). From Brain Organoids to Networking Assembloids: Implications for Neuroendocrinology and Stress Medicine. *Front. Physiol.* 12:621970. 10.3389/fphys.2021.621970 34177605 PMC8222922

[B59] Mansour A. A. Gonçalves J. T. Bloyd C. W. Li H. Fernandes S. Quang D. (2018). An in vivo model of functional and vascularized human brain organoids. *Nat. Biotechnol.* 36 432–441. 10.1038/nbt.4127 29658944 PMC6331203

[B60] Miura Y. Li M. Y. Birey F. Ikeda K. Revah O. Thete M. V. (2020). Generation of human striatal organoids and cortico-striatal assembloids from human pluripotent stem cells. *Nat. Biotechnol.* 38 1421–1430. 10.1038/s41587-020-00763-w 33273741 PMC9042317

[B61] Muguruma K. Nishiyama A. Kawakami H. Hashimoto K. Sasai Y. (2015). Self-organization of polarized cerebellar tissue in 3D culture of human pluripotent stem cells. *Cell. Rep.* 10 537–550. 10.1016/j.celrep.2014.12.051 25640179

[B62] Nascimento J. M. Saia-Cereda V. M. Sartore R. C. da Costa R. M. Schitine C. S. Freitas H. R. (2019). Human Cerebral Organoids and Fetal Brain Tissue Share Proteomic Similarities. *Front. Cell. Dev. Biol.* 7:303. 10.3389/fcell.2019.00303 31850342 PMC6893972

[B63] Nguyen D. Smith L. M. Lagasse L. L. Derauf C. Grant P. Shah R. (2010). Intrauterine growth of infants exposed to prenatal methamphetamine: results from the infant development, environment, and lifestyle study. *J. Pediatr.* 157 337–339. 10.1016/j.jpeds.2010.04.024 20570284 PMC3018351

[B64] O’Connor M. J. Paley B. (2009). Psychiatric conditions associated with prenatal alcohol exposure. *Dev. Disabil. Res. Rev.* 15 225–234. 10.1002/ddrr.74 19731386

[B65] Padula A. M. Monk C. Brennan P. A. Borders A. Barrett E. S. McEvoy C. T. (2020). A review of maternal prenatal exposures to environmental chemicals and psychosocial stressors-implications for research on perinatal outcomes in the ECHO program. *J. Perinatol.* 40 10–24. 10.1038/s41372-019-0510-y 31616048 PMC6957228

[B66] Pamies D. Block K. Lau P. Gribaldo L. Pardo C. A. Barreras P. (2018). Rotenone exerts developmental neurotoxicity in a human brain spheroid model. *Toxicol. Appl. Pharmacol.* 354 101–114. 10.1016/j.taap.2018.02.003 29428530 PMC6082736

[B67] Parasuraman S. (2011). Toxicological screening. *J. Pharmacol. Pharmacother.* 2 74–79. 10.4103/0976-500x.81895 21772764 PMC3127354

[B68] Paşca S. P. (2018). The rise of three-dimensional human brain cultures. *Nature* 553 437–445. 10.1038/nature25032 29364288

[B69] Pellegrini L. Albecka A. Mallery D. L. Kellner M. J. Paul D. Carter A. P. (2020a). SARS-CoV-2 Infects the Brain Choroid Plexus and Disrupts the Blood-CSF Barrier in Human Brain Organoids. *Cell Stem Cell* 27 951.e–961.e. 10.1016/j.stem.2020.10.001 33113348 PMC7553118

[B70] Pellegrini L. Bonfio C. Chadwick J. Begum F. Skehel M. Lancaster M. A. (2020b). Human CNS barrier-forming organoids with cerebrospinal fluid production. *Science* 369 eaaz5626. 10.1126/science.aaz5626 32527923 PMC7116154

[B71] Pham M. T. Pollock K. M. Rose M. D. Cary W. A. Stewart H. R. Zhou P. (2018). Generation of human vascularized brain organoids. *Neuroreport* 29 588–593. 10.1097/wnr.0000000000001014 29570159 PMC6476536

[B72] Qian X. Jacob F. Song M. M. Nguyen H. N. Song H. Ming G. L. (2018). Generation of human brain region-specific organoids using a miniaturized spinning bioreactor. *Nat. Protoc.* 13 565–580. 10.1038/nprot.2017.152 29470464 PMC6241211

[B73] Qian X. Nguyen H. N. Song M. M. Hadiono C. Ogden S. C. Hammack C. (2016). Brain-Region-Specific Organoids Using Mini-bioreactors for Modeling ZIKV Exposure. *Cell* 165 1238–1254. 10.1016/j.cell.2016.04.032 27118425 PMC4900885

[B74] Qian X. Su Y. Adam C. D. Deutschmann A. U. Pather S. R. Goldberg E. M. (2020). Sliced Human Cortical Organoids for Modeling Distinct Cortical Layer Formation. *Cell Stem Cell* 26 766.e–781.e. 10.1016/j.stem.2020.02.002 32142682 PMC7366517

[B75] Qiao H. Zhang Y. S. Chen P. (2018). Commentary: Human brain organoid-on-a-chip to model prenatal nicotine exposure. *Front. Bioeng. Biotechnol.* 6:138. 10.3389/fbioe.2018.00138 30338258 PMC6180184

[B76] Quadrato G. Nguyen T. Macosko E. Z. Sherwood J. L. Min Yang S. Berger D. R. (2017). Cell diversity and network dynamics in photosensitive human brain organoids. *Nature* 545 48–53. 10.1038/nature22047 28445462 PMC5659341

[B77] Rando K. Chaplin T. M. Potenza M. N. Mayes L. Sinha R. (2013). Prenatal cocaine exposure and gray matter volume in adolescent boys and girls: relationship to substance use initiation. *Biol. Psychiatry.* 74 482–489. 10.1016/j.biopsych.2013.04.030 23751204 PMC3775853

[B78] Renner H. Becker K. J. Kagermeier T. E. Grabos M. Eliat F. Günther P. (2021). Cell-Type-Specific High Throughput Toxicity Testing in Human Midbrain Organoids. *Front. Mol. Neurosci.* 14:715054. 10.3389/fnmol.2021.715054 34335182 PMC8321240

[B79] Riley E. P. McGee C. L. (2005). Fetal alcohol spectrum disorders: an overview with emphasis on changes in brain and behavior. *Exp. Biol. Med.* 230 357–365. 10.1177/15353702-0323006-03 15956765

[B80] Rowe R. G. Daley G. Q. (2019). Induced pluripotent stem cells in disease modelling and drug discovery. *Nat. Rev. Genet.* 20 377–388. 10.1038/s41576-019-0100-z 30737492 PMC6584039

[B81] Sandström J. Eggermann E. Charvet I. Roux A. Toni N. Greggio C. (2017). Development and characterization of a human embryonic stem cell-derived 3D neural tissue model for neurotoxicity testing. *Toxicol. In. Vitro.* 38 124–135. 10.1016/j.tiv.2016.10.001 27729293

[B82] Seiler A. E. Spielmann H. (2011). The validated embryonic stem cell test to predict embryotoxicity in vitro. *Nat. Protoc.* 6 961–978. 10.1038/nprot.2011.348 21720311

[B83] Shi Y. Sun L. Wang M. Liu J. Zhong S. Li R. (2020). Vascularized human cortical organoids (vOrganoids) model cortical development in vivo. *PLoS Biol.* 18:e3000705. 10.1371/journal.pbio.3000705 32401820 PMC7250475

[B84] Singer L. T. Arendt R. Minnes S. Farkas K. Salvator A. Kirchner H. L. (2002). Cognitive and motor outcomes of cocaine-exposed infants. *Jama* 287 1952–1960. 10.1001/jama.287.15.1952 11960537 PMC10246332

[B85] Sirenko O. Parham F. Dea S. Sodhi N. Biesmans S. Mora-Castilla S. (2019). Functional and Mechanistic Neurotoxicity Profiling Using Human iPSC-Derived Neural 3D Cultures. *Toxicol. Sci.* 167 58–76. 10.1093/toxsci/kfy218 30169818 PMC6317428

[B86] Sivitilli A. A. Gosio J. T. Ghoshal B. Evstratova A. Trcka D. Ghiasi P. (2020). Robust production of uniform human cerebral organoids from pluripotent stem cells. *Life Sci. Alliance* 3:e202000707. 10.26508/lsa.202000707 32303588 PMC7167289

[B87] Smart I. H. Dehay C. Giroud P. Berland M. Kennedy H. (2002). Unique morphological features of the proliferative zones and postmitotic compartments of the neural epithelium giving rise to striate and extrastriate cortex in the monkey. *Cereb. Cortex.* 12 37–53. 10.1093/cercor/12.1.37 11734531 PMC1931430

[B88] Smith L. M. LaGasse L. L. Derauf C. Grant P. Shah R. Arria A. (2006). The infant development, environment, and lifestyle study: effects of prenatal methamphetamine exposure, polydrug exposure, and poverty on intrauterine growth. *Pediatrics* 118 1149–1156. 10.1542/peds.2005-2564 16951010

[B89] Sun D. (2016). The potential of neural transplantation for brain repair and regeneration following traumatic brain injury. *Neural. Regen. Res.* 11 18–22. 10.4103/1673-5374.169605 26981070 PMC4774215

[B90] Sun N. Meng X. Liu Y. Song D. Jiang C. Cai J. (2021). Applications of brain organoids in neurodevelopment and neurological diseases. *J. Biomed. Sci.* 28:30. 10.1186/s12929-021-00728-4 33888112 PMC8063318

[B91] Takahashi K. Tanabe K. Ohnuki M. Narita M. Ichisaka T. Tomoda K. (2007). Induction of pluripotent stem cells from adult human fibroblasts by defined factors. *Cell* 131 861–872. 10.1016/j.cell.2007.11.019 18035408

[B92] Takahashi T. (2019). Organoids for Drug Discovery and Personalized Medicine. *Annu. Rev. Pharmacol. Toxicol.* 59 447–462. 10.1146/annurev-pharmtox-010818-021108 30113875

[B93] Tao Y. Vermilyea S. C. Zammit M. Lu J. Olsen M. Metzger J. M. (2021). Autologous transplant therapy alleviates motor and depressive behaviors in parkinsonian monkeys. *Nat. Med.* 27 632–639. 10.1038/s41591-021-01257-1 33649496 PMC8198752

[B94] Tao Y. Zhang S. C. (2016). Neural Subtype Specification from Human Pluripotent Stem Cells. *Cell Stem Cell* 19 573–586. 10.1016/j.stem.2016.10.015 27814479 PMC5127287

[B95] Truskey G. A. (2018). Human Microphysiological Systems and Organoids as in Vitro Models for Toxicological Studies. *Front. Pub. Health* 6:185. 10.3389/fpubh.2018.00185 30042936 PMC6048981

[B96] Wang H. (2018). Modeling Neurological Diseases With Human Brain Organoids. *Front Synaptic Neurosci* 10:15. 10.3389/fnsyn.2018.00015 29937727 PMC6002496

[B97] Wang Y. Chiola S. Yang G. Russell C. Armstrong C. J. Wu Y. (2021). Modeling autism-associated SHANK3 deficiency using human cortico-striatal organoids generated from single neural rosettes. *bioRxiv* 2021.2001.2025.428022, 10.1101/2021.01.25.428022 PMC9537523 36202854

[B98] Wang Y. Wang L. Guo Y. Zhu Y. Qin J. (2018). Engineering stem cell-derived 3D brain organoids in a perfusable organ-on-a-chip system. *RSC adv.* 8 1677–1685. 10.1039/c7ra11714k PMC9077091 35540867

[B99] Wang Z. Wang S. N. Xu T. Y. Hong C. Cheng M. H. Zhu P. X. (2020). Cerebral organoids transplantation improves neurological motor function in rat brain injury. *CNS Neurosci. Ther.* 26 682–697. 10.1111/cns.13286 32087606 PMC7298981

[B100] Xiang Y. Cakir B. Park I. H. (2020). Deconstructing and reconstructing the human brain with regionally specified brain organoids. *Semin. Cell Dev. Biol*. 111 40–51. 10.1016/j.semcdb.2020.05.023 32553582 PMC12581431

[B101] Xiang Y. Tanaka Y. Cakir B. Patterson B. Kim K. Y. Sun P. (2019). hESC-Derived Thalamic Organoids Form Reciprocal Projections When Fused with Cortical Organoids. *Cell Stem Cell* 24 487.e–497.e. 10.1016/j.stem.2018.12.015 30799279 PMC6853597

[B102] Xiang Y. Tanaka Y. Patterson B. Kang Y. J. Govindaiah G. Roselaar N. (2017). Fusion of Regionally Specified hPSC-Derived Organoids Models Human Brain Development and Interneuron Migration. *Cell Stem Cell* 21 383.e–398.e. 10.1016/j.stem.2017.07.007 28757360 PMC5720381

[B103] Yamasue H. Domes G. (2018). Oxytocin and Autism Spectrum Disorders. *Curr. Top Behav. Neurosci.* 35 449–465. 10.1007/7854_2017_24 28766270

[B104] Yin X. Mead B. E. Safaee H. Langer R. Karp J. M. Levy O. (2016). Engineering Stem Cell Organoids. *Cell Stem Cell* 18 25–38. 10.1016/j.stem.2015.12.005 26748754 PMC4728053

[B105] Yu J. Vodyanik M. A. Smuga-Otto K. Antosiewicz-Bourget J. Frane J. L. Tian S. (2007). Induced pluripotent stem cell lines derived from human somatic cells. *Science* 318 1917–1920. 10.1126/science.1151526 18029452

[B106] Zhu Y. Wang L. Yin F. Yu Y. Wang Y. Shepard M. J. (2017). Probing impaired neurogenesis in human brain organoids exposed to alcohol. *Integr. Biol.* 9 968–978. 10.1039/c7ib00105c 29168871

